# To Do or Not to Do; Dilemma of Intra-Arterial Revascularization in Acute Ischemic Stroke

**DOI:** 10.1371/journal.pone.0099261

**Published:** 2014-06-06

**Authors:** Joon-Tae Kim, Suk-Hee Heo, Ji Sung Lee, Myeong-Ho Park, Dong-Seok Oh, Kang-Ho Choi, Ihn-Gyu Kim, Yeon Soo Ha, Hyuk Chang, In Sung Choo, Seong Hwan Ahn, Seul-Ki Jeong, Byoung-Soo Shin, Man-Seok Park, Ki-Hyun Cho

**Affiliations:** 1 Department of Neurology, Cerebrovascular Center, Chonnam National University Hospital, Gwangju, Korea; 2 Department of Radiology, Chonnam National University Hwasun Hospital, Hwasun, Korea; 3 Biostatistical Consulting Unit, Soonchunhyang University Medical Center, Seoul, Korea; 4 Department of Neurology, Wonkwang University Hospital, Iksan, Korea; 5 Department of Neurology, Chosun University Hospital, Gwangju, Korea; 6 Department of Neurology, Chonbuk National University Medical School and Hospital, Jeonju, Korea; Cuban Neuroscience Center, Cuba

## Abstract

**Background:**

There has still been lack of evidence for definite imaging criteria of intra-arterial revascularization (IAR). Therefore, IAR selection is left largely to individual clinicians. In this study, we sought to investigate the overall agreement of IAR selection among different stroke clinicians and factors associated with good agreement of IAR selection.

**Methods:**

From the prospectively registered data base of a tertiary hospital, we identified consecutive patients with acute ischemic stroke. IAR selection based on the provided magnetic resonance imaging (MRI) results and clinical information were independently performed by 5 independent stroke physicians currently working at 4 different university hospitals. MRI results were also reviewed by 2 independent experienced neurologists blinded to clinical data and physicians' IAR selection. The Alberta Stroke Program Early Computed Tomography Score (ASPECTS) was calculated on initial DWI and MTT. We arbitrarily used ASPECTS differences between DWI and MTT (D-M ASPECTS) to quantitatively evaluate mismatch.

**Results:**

The overall interobserver agreement of IAR selection was fair (kappa = 0.398). In patients with DWI-ASPECTS >6, interobserver agreement was moderate to substantial (0.398–0.620). In patients with D-M ASPECTS >4, interobserver agreement was moderate to almost perfect (0.532–1.000). Patients with higher DWI or D-M ASPECTS had better agreement of IAR selection.

**Conclusion:**

Our study showed that DWI-ASPSECTS >6 and D-M ASPECTS >4 had moderate to substantial agreement of IAR selection among different stroke physicians. However, there is still poor agreement as to whether IAR should not be performed in patients with lower DWI and D-M ASPECTS.

## Introduction

Intra-arterial revascularization (IAR) has been performed in large stroke centers and may provide higher rates of recanalization than placebo or intravenous thrombolysis (IVT) [1], [2]. Because IVT is least efficacious in patients with proximal arterial occlusion and clinically severe stroke, additional IAR is considered a promising therapeutic option [3]. Previous observational studies showed that IAR, including mechanical thrombectomy, has a high recanalization rate and a good prognosis [2], [4]. With advances in devices, such as mechanical clot disruption, coil retrievers, suction apparatus, and stent retrievers [5]–[9], IAR techniques have recently been improved remarkably, showing higher rates of recanalization [8], [9].

However, recent clinical trials on IAR failed to demonstrate the beneficial effects of IAR in patients with acute ischemic stroke (AIS) [10]–[12]. It should be cautiously interpreted because recent trials have many shortcomings in that many patients did not have the large vessel occlusion with sufficient salvageable tissues [13]. Due to these negative results, it is difficult to determine which patients should be treated with IAR in clinical practice. Currently, IAR is recommended that carefully selected patients with major ischemic stroke within 6–8 hours of symptom onset who are not otherwise candidates for IVT (Class I; Level of Evidence B) [14].

As magnetic resonance imaging (MRI) has been considered an important imaging tool for the diagnosis of AIS, MRI-based IAR has been increasingly performed in large centers. In addition, lesion volume measured by using diffusion-weighted imaging (DWI) and the DWI-Alberta Stroke Program Early Computed Tomography Score (ASPECTS) is known to be a good tool for predicting functional outcomes in AIS [15], [16]. Thus, treatment options selected on the basis of these results may help improve clinical outcomes. Although pretreatment MRI has proved to be safe and feasible for thrombolysis [16], there has still been lack of definite imaging criteria for IAR [17]. In clinical practice, it is still unclear whether IAR has been performed only in patients whose prognoses are expected to be good. IAR decision is therefore left largely to individual clinicians.

It would be important to evaluate how many proportions of physicians reach concordance about IAR decision in the same patients. To improve the agreement level in clinical practice, it might be helpful to know which factors were associated with high agreement of IAR decision. In addition, comparisons of clinical outcomes according to the agreement of IAR decision could predict the effect and accuracy of IAR decision.

In this study, we sought to investigate the overall agreement of IAR decision using MRI of the same patients among different stroke clinicians as well as factors associated with good (or poor) agreement of IAR decision. In addition, we compared the clinical outcomes between patients who underwent IAR and those who did not, according to the decision of physicians.

## Methods

### 1. Subjects

From the prospectively registered data base of the Cerebrovascular Center of Chonnam National University Hospital, we screened 955 consecutive patients with acute ischemic stroke admitted to our hospital between January 2012 and January 2013. One hundred sixty-seven patients were those who (1) presented within 6 hours after first known abnormal time; (2) underwent emergency MRI; and (3) had symptomatic middle cerebral artery or internal carotid artery occlusion. We excluded 42 patients who had (1) multiple territorial lesions, such as bilateral lesions or both supratentorial and infratentorial lesions (N = 15); (2) other etiologies, such as Moyamoya disease (N = 3); (3) incomplete imaging such as no perfusion weighted imaging (PWI) or fluid-attenuated inversion recovery (FLAIR) (N = 17) and (4) no diffusion weighted imaging (DWI) lesions (N = 7) (Figure S1).

### 2. Ethics Statement

This study was approved by the Institutional Review Board (IRB) of Chonnam National University Hospital. Written informed consent was not obtained from participants because of the retrospective design of this study; therefore, the IRB of the hospital waived the need for written informed consent from participants.

### 3. IAR decision

IAR decision based on the provided MRI and clinical information was independently conducted by 5 stroke physicians currently working at 4 different university hospitals in the Honam area, Republic of Korea. We used the internet clouding system to upload the MRI (Digital Imaging and Communications in Medicine, DICOM files) and clinical information of the patients. All physicians downloaded DICOM files of MRI and clinical information and independently determined whether each patient would undergo IAR. Thereafter, they sent their IAR decision to a single physician (M.H.P.) at the central laboratory who was not included in the decision process. The clinical information provided to physicians were as follows; age, gender, initial National Institutes of Health Stroke Scale (NIHSS) scores, clinical manifestations, the interval between symptom onset and hospital visit, time from symptom onset to MRI (first known abnormal time to MRI in patients with unclear onset), clear/unclear onset, and risk factors. Patients' private information, such as names, and ID numbers, were not included. MRI sequences including initial DWI, FLAIR, intra- and extracranial time of flight MR angiography, and PWI (color maps of mean transit time [MTT] and cerebral blood volume) were provided. In order to assess intra-rater reliability, repeat measurements were made on 20 randomly selected patients 2 months after the first IAR decision, and physicians were blinded to information on repeat measurements.

### 4. Imaging analysis

MRI results were retrospectively reviewed by 2 experienced neurologists (K.H. C.and M.S. P.) in a core imaging laboratory blinded to clinical data and physicians' IAR decision. The ASPECTS was calculated on initial DWI and MTT [18], [19]. We arbitrarily used ASPECTS differences between DWI and MTT (DWI-ASPECTS minus MTT-ASPECTS, D-M ASPECTS) to quantitatively evaluate mismatch. Arterial occlusion sites relevant to ischemia were determined by analysis of the initial MRA, which included the proximal and distal internal carotid artery (ICA) and the M1/M2 segment of the middle cerebral artery (MCA). Based on FLAIR imaging results, periventricular white matter hyperintensities were graded from 0 to 3 on Fazeka's scale, with scores of 2 and 3 considered severe periventricular white matter hyperintensities [20]. The presence of previous ischemic lesions and frank hyperintensity on FLAIR images were assessed as well. Discrepancy was resolved by consensus conference.

### 5. Clinical assessment

We collected data of functional outcomes at 3 months using the modified Rankin Scale (mRS) scores. The mRS of 0–2 at 90 days was defined as favorable outcomes. Mortality was defined as death within 3months.

### 6. Statistical analysis

Kappa statistics for multiple raters were calculated to assess inter–rater agreement using the magree macro in SAS version 9.2 (SAS Institute, Cary, NC, USA). The agreement level was rated as slight (0–0.2), fair (0.2–0.4), moderate (0.4–0.6), substantial (0.6–0.8), or almost perfect (0.8–1.0) [21]. Intra-observer reliability was calculated by simple kappa statistics.

**Figure 1:**
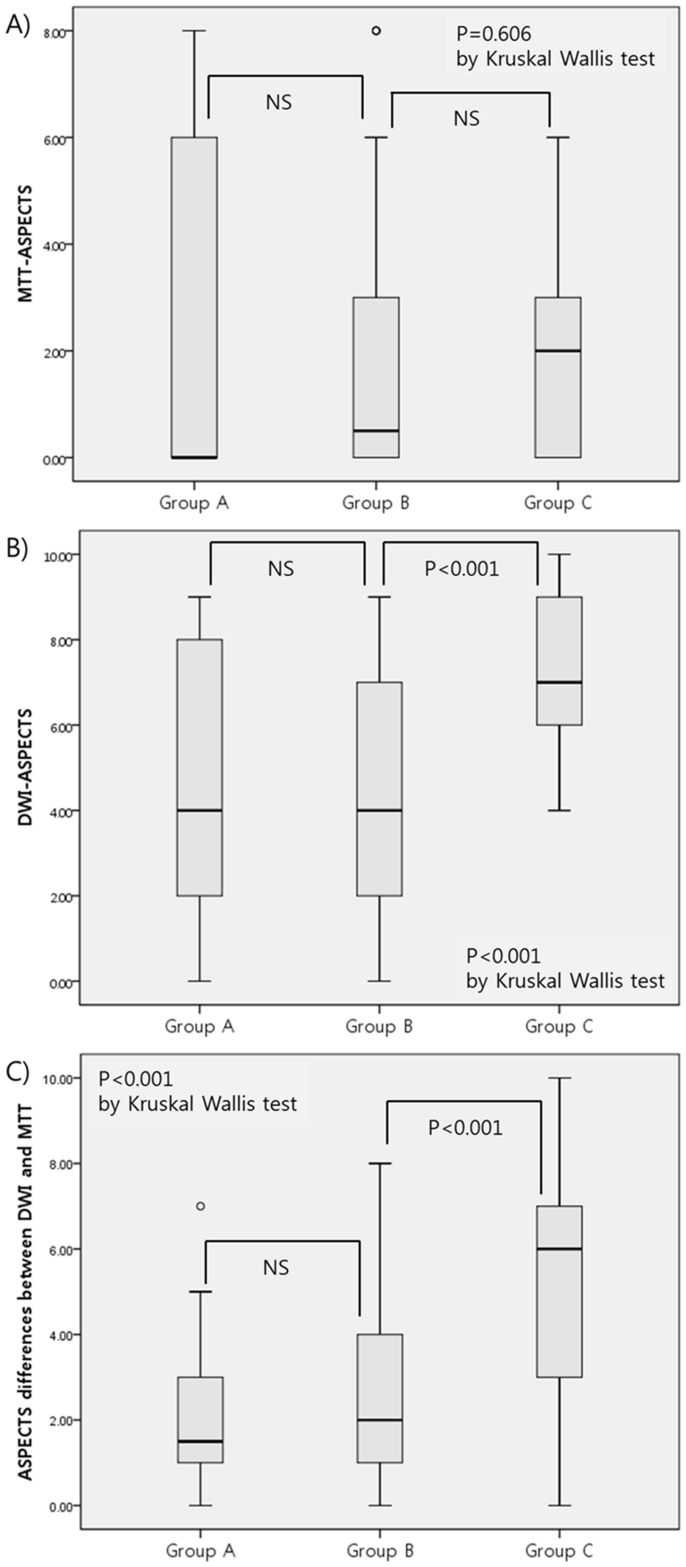
Representative cases of lesion patterns of poor agreements for IAR (A, group B) and lesion patterns with good agreement for IAR decision (B, group C). (A) The figures show moderate sized lesions of right hemisphere on DWI, occlusion of right distal internal carotid artery, and large perfusion deficit on PWI. (B) The figures show small lesion of basal ganglia on DWI, occlusion of right internal carotid artery, and large hemispheric perfusion deficit on PWI.

The percentage, mean (±SD), or median (interquartile range, IQR) are presented depending on variable characteristics. Patients were divided into 3 groups according to the proportion of physicians for IAR selection: those on whom none or 1 of the 5 physicians agreed to perform IAR (group A), those on whom 2 or 3 of the 5 physicians agreed to perform IAR (group B, figure 1-A), and those on whom 4 or all of the 5 physicians agreed to perform IAR (group C, figure 1-B). Categorical variables were analyzed using the χ^2^-test (or Fisher's exact test as appropriate) between individual groups. Continuous variables were analyzed using the independent samples *t* test (or the Mann–Whitney *U* test as appropriate) between individual groups and one-way ANOVA test (or Kruskall-Wallis test) between the 3 groups. A *p* value of <0.05 was considered statistically significant. All of the statistical analyses were performed using SPSS for Windows, version 17 (SPSS Inc., Chicago, IL, USA).

## Results

### 1. General Characteristics

A total of 125 patients with AIS and symptomatic MCA or ICA occlusion were analyzed in this study. The general characteristics of the 125 patients (mean age, 69.3±12.8 years) are shown in Table 1. Median NIHSS scores were 12.0 (IQR, 9.0). Seven of the 24 patients with occlusion in M2 or more distal segments had catheter-inaccessible M2 or M3 segments. Patients with occlusion of catheter-inaccessible M2 or M3 segments (n = 7) had a DWI-ASPECTS of 8 (IQR, 1.0) and a D-M ASPECTS of 0 (IQR, 2.0). The general characteristics of the 5 stroke physicians are shown in Table S1. They were dedicated stroke physicians working at 4 stroke centers of local university hospitals. Since Chonnam National University Hospital is the largest one in this area, 2 physicians (J.T.K., D.S.O.) participated at the decision process.

**Table 1:** The general characteristics of the subjects.

Characteristics	Subjects (N = 125)
Age (mean±SD)	69.27±12.75
Male (N, %)	72 (57.6)
Risk factors (n, %)	
Hypertension	84 (67.2)
Diabetes mellitus	22 (17.6)
Atrial fibrillation	55 (44.0)
Dyslipidemia	32 (25.6)
Smoking	25 (20.0)
Previous stroke	14 (11.2)
Coronary artery disease	12 (9.6)
Occlusion sites (n, %)	
ICA	57 (45.6)
Distal ICA	7 (5.6)
M1	37 (29.6)
M2 and more distal	24 (19.2)
M2 distal	7 (5.6)
Baseline NIHSS (med, IQR)	12 (9.0)
Cortical symptoms (n, %)	93 (74.4)
NIHSS 0–4 (n, %)	21 (16.8)
FAT to visit (minutes; mean±SD)	186.96±83.94
IVT (n, %)	58 (46.4)
IAR (n, %)	36 (28.8)

Abbreviations: ICA; internal carotid artery, M1; M1 segment of middle cerebral artery, M2; M2 segment of middle cerebral artery, FAT; first known abnormal time, IVT; intravenous thrombolysis, IAR; intra-arterial revascularization.

### 2. Interobserver agreement of IAR decision

The overall interobserver agreement of IAR decision according to both MRI results and clinical information was fair (kappa = 0.398). Interobserver agreement of IAR decision analyzed by the DWI-ASPECTS, MTT-ASPECTS and D-M ASPECTS is shown in Table 2. Based on DWI-ASPECTS, interobserver agreement of IAR decision in patients with DWI-ASPECTS ≤5 was poor. However, in patients with DWI -ASPECTS >6, the interobserver agreement was moderate to substantial (kappa; 0.457–0.620 for a DWI-ASPECT of 7–10). Similarly, poor agreement was found in patients with D-M ASPECTS <5 except for patients with a D-M ASPECTS of 3. In patients with D-M ASPECTS >7, interobserver agreement was substantial to almost perfect (kappa = 0.626–1.000 for a D-M ASPECTS 8–10). Patients with a higher D-M ASPECTS had better agreement of IAR decision. However, in patients with MTT-ASPECTS >4 except for patients with a MTT-ASPECTS of 6, interobserver agreement was poor.

**Table 2:** Interobserver agreement of IAR selection between 5 clinicians for 125 stroke patients.

DWI ASPECTS			MTT ASPECTS			D-M ASPECTS		
Scores	N (%)	k value	Scores	N (%)	k value	Scores	N (%)	k value
0	6 (4.8)	−0.042	0	47 (37.6)	0.353	0	20 (16.0)	0.033
1	5 (4.0)	−0.103	1	12 (9.6)	0.246	1	15 (12.0)	0.190
2	6 (4.8)	0.025	2	14 (11.2)	0.409	2	14 (11.2)	0.183
3	5 (4.0)	−0.122	3	20 (16.0)	0.405	3	13 (10.4)	0.439
4	9 (7.2)	−0.184	4	4 (3.2)	0.688	4	10 (8.0)	0.064
5	6 (4.8)	−0.200	5	3 (2.4)	−0.050	5	11 (8.8)	0.532
6	14 (11.2)	0.398	6	7 (5.6)	0.345	6	14 (11.2)	0.419
7	19 (15.2)	0.457	7	4 (3.2)	0.020	7	11 (8.8)	0.532
8	21 (16.8)	0.478	8	13 (10.4)	0.129	8	7 (5.6)	0.626
9	20 (16.0)	0.586	9	1 (0.8	−	9	5 (4.0)	0.781
10	14 (11.2)	0.620	10	0	−	10	5 (4.0)	1.000
>5	88 (70.4)	0.554	<5	97 (77.6)	0.373	>5	42 (33.6)	0.598
>6	74 (59.2)	0.578	<4	93 (74.4)	0.386	>6	28 (22.4)	0.694
>7	55 (44.0)	0.585	<3	73 (58.4)	0.372	>7	17 (13.6)	0.802

The level of agreement is represented by kappa (κ) value calculated using DWI, MTT, and D-M ASPECTS.

Abbreviations: DWI; diffusion-weighted imaging, ASPECTS; Alberta Stroke Program Early Computed Tomography Score, MTT; mean transit time, D-M ASPECTS; ASPECTS differences between DWI and MTT.

### 3. Comparisons of characteristics between groups B and A/C

Comparisons of general characteristics between the 3 groups are shown in Table 3. The frequency of NIHSS <5 was significantly higher in group A than in groups B and C (51.3% versus 5.3% and 0%, *p*<0.001 for each). There were 31 patients with unclear onset but only 6 of them were found not to perform IAR by any physicians, and the frequency of unclear onset was significantly different between groups B and C/A. Groups A and C had higher DWI-ASPECTS than group B (median; 8.0 and 7.0 versus 4.0, *p*<0.001 for each). Group C also had a higher D-M ASPECTS than group B (median 6.0 versus 2.0, *p*<0.001). MTT-ASPECTS were not significantly different between the 3 groups.

**Table 3:** The general characteristics of the 3 groups according to the proportion of physicians who selected IAR.

	Group A (N = 37)	Group B (N = 38)	Group C (N = 50)	P1 (A vs B)	P2 (B vs C)	P3 (trends)
Age (mean±SD)	67.48±16.24	71.84±12.23	68.64±9.84	0.195	0.177	0.305
Male (N, %)	21 (56.8)	23 (60.5)	28 (56.0)	0.817	0.828	0.915
Risk factors (n, %)						
Hypertension	25 (67.6)	27 (71.1)	32 (64.0)	0.805	0.504	0.691
Diabetes mellitus	5 (13.5)	6 (15.8)	11 (22.0)	>0.999	0.589	0.294
Atrial fibrillation	12 (32.4)	20 (52.6)	23 (46.0)	0.103	0.667	0.252
Dyslipidemia	11 (29.7)	7 (18.4)	14 (28.0)	0.289	0.325	0.936
Smoking	6 (16.2)	11 (28.9)	8 (16.0)	0.271	0.192	0.872
Previous stroke	4 (10.8)	7 (18.4)	3 (6.0)	0.516	0.093	0.402
Coronary artery disease	1 (2.7)	5 (13.2)	6 (12.0)	0.200	>0.999	0.170
FAT to visit (minutes; mean±SD)	179.2±102.4	209.3±66.9	175.7±78.6	0.137	0.037	0.142
Unclear onset (n, %)	10 (27.0)	8 (21.1)	13 (26.0)	0.597	0.624	0.955
NIHSS (med, IQR)	3.0 (11.5)	14.0 (8.0)	13.0 (5.25)	<0.001	0.888	<0.001
NIHSS ≤4 (n, %)	19 (51.3)	2 (5.3)	0	<0.001	0.184	<0.001
Cortical symptoms (n, %)	15 (40.5)	33 (86.8)	45 (90.0)	<0.001	0.740	<0.001
FLAIR change (n, %)	5 (13.5)	6 (15.8)	5 (10.0)	>0.999	0.520	0.592
Severe PVWMH (n, %)	12 (32.4)	11 (28.9)	12 (24.0)	0.805	0.631	0.383
Previous lesions (n, %)	7 (18.9)	17 (44.7)	11 (22.0)	0.025	0.037	0.931
Occlusion sites (n, %)				0.488	0.003	0.002
ICA occlusion	14 (37.8)	21 (55.3)	22 (44.0)			
dICA occlusion	2 (5.4)	2 (5.3)	3 (6.0)			
M1 occlusion	9 (24.3)	6 (15.8)	23 (46.0)			
M2 occlusion	13 (35.1)	9 (23.7)	2 (4.0)			
DWI-ASPECTS (med, IQR)	8.0 (3.0)	4.0 (6.0)	7.0 (3.0)	0.002	<0.001	<0.001
>6 (n, %)	26 (70.3)	13 (34.2)	35 (70.0)	0.003	0.001	0.775
MTT-ASPECTS (med, IQR)	3.0 (7.5)	1.0 (4.25)	2.0 (3.0)	0.096	0.340	0.139
D-M ASPECTS (med, IQR)	2.0 (5.0)	2.0 (4.0)	6.0 (4.25)	0.138	0.001	<0.001
>6 (n, %)	7 (18.9)	2 (5.3)	19 (38.0)	0.086	<0.001	0.019

Abbreviations: FAT; first known abnormal time, FLAIR; Fluid-attenuated inversion recovery, PVWMH; periventricular white matter hyperintensity, M2; M2 segment of middle cerebral artery. ICA; internal carotid artery, dICA; distal ICA, M1; M1 segment of middle cerebral artery, M2; M2 segment of middle cerebral artery, DWI; diffusion-weighted imaging, ASPECTS; Alberta Stroke Program Early Computed Tomography Score, MTT; mean transit time, D-M ASPECTS; ASPECTS differences between DWI and MTT.

**Figure 2:**
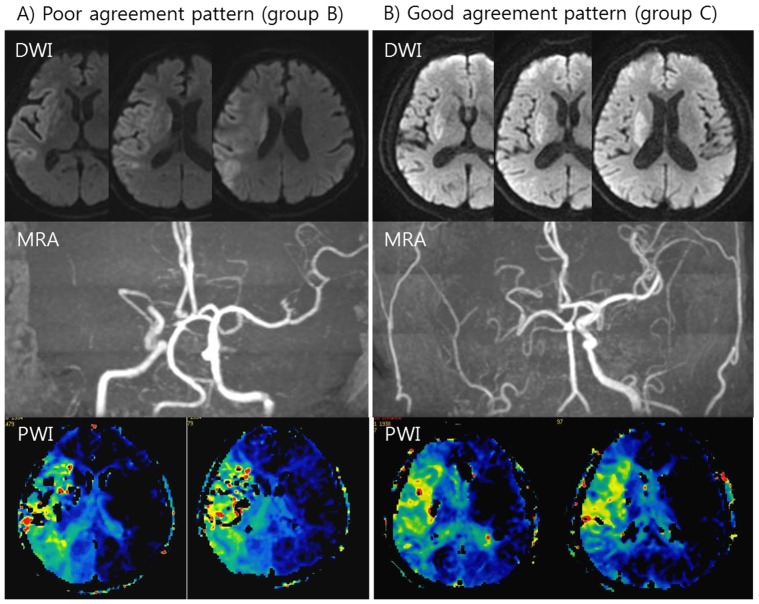
Comparisons of imaging characteristics between groups B and A/C (N = 98; exclusion of patients with NIHSS <5 and occlusion of distal M2 segments). (A) MTT-ASPECTS, (B) DWI-ASPECTS, and (C) ASPECTS differences between DWI and MTT.

In patients (n = 98) excluding those with NIHSS <5 and occlusion of catheter inaccessible M2 and M3 segments, imaging characteristics among groups are shown in Figure 2. Unlike Table 3, group A had a median DWI-ASPECTS of 4 and a median D-M ASPECTS of 1.75. Patients with IAR decision had significantly higher DWI-ASPECTS and D-M ASPECTS (*p* for the trend, <0.001 for each). In addition, the DWI-ASPECTS and the D-M ASPECTS were significantly different between groups B and C, but not between groups B and A (Figure 2).

### 4. Intra-observer reliability

Intra-observer reliability was analyzed by simple kappa statistics. Intra-observer reliability of each physician was good (I.S.C., D.S.O., J.T.K., S.K.J., and Y.S.H.; kappa = 0.802, 0.800, 0.794, 0.700, and 0.694, from the highest to the lowest value).

### 5. Clinical outcomes

In group C (n = 50), IAR was performed on 29 patients (58%) in the real clinical setting. Of these 29 patients, 11 (37.9%) had favorable outcomes at 3 months, and there was no death within 3 months. IAR was independently associated with favorable outcomes at 3months, adjusted by age and baseline NIHSS scores (OR, 6.059; 95% CI, 1.003–36.583; p = 0.046). In contrast, of the 21 patients who did not undergo IAR, only 2 (9.5%) had favorable outcomes at 3 months, and 6 (28.6%) died within 3months (Table 4). The number of patients who underwent IAR was very small in groups A and B (4/37 and 3/38, respectively).

**Table 4:** Comparisons of outcomes according to IAR treatment in patients of each group.

	Group A			Group B			Group C		
	No IAR (N = 33)	IAR (N = 4)	p	No IAR (N = 35)	IAR (N = 3)	p	No IAR (N = 21)	IAR (N = 29)	p
Age (mean, SD)	68.9±14.8	55.8±25.2	0.283	71.9±11.9	70.3±19.2	0.879	69.9±9.4	67.7±10.2	0.523
Male (n, %)	17 (51.5)	4 (100)	0.118	22 (62.9)	1 (33.3)	0.550	14 (66.7)	14 (48.3)	0.254
Risk factors (n, %)									
Hypertension	23 (69.7)	2 (50.0)	0.582	24 (68.6)	3 (100)	0.542	15 (71.4)	17 (58.6)	0.388
Diabetes	3 (9.1)	2 (50.0)	0.080	6 (17.1)	0	>0.99	6 (28.6)	5 (17.2)	0.491
Atrial fibrillation	12 (36.4)	0	0.282	19 (54.3)	1 (33.3)	0.595	10 (47.6)	13 (44.8)	>0.99
Dyslipidemia	10 (30.3)	1 (25.0)	>0.99	7 (20.0)	0	>0.99	7 (33.3)	7 (24.1)	0.534
Unclear onset (n, %)	8 (24.2)	2 (50.0)	0.291	8 (22.9)	0	>0.99	4 (19.0)	9 (31.0)	0.515
FLAIR changes (n, %)	4 (12.1)	1 (25.0)	0.456	5 (14.3)	1 (33.3)	0.412	1 (4.8)	4 (13.8)	0.383
ICA occlusion (n, %)	13 (39.4)	3 (75.0)	0.296	21 (60.0)	2 (66.7)	>0.99	12 (57.1)	13 (44.8)	0.567
NIHSS (med, IQR)	4 (13.5)	2 (2.25)	0.082	13 (8.0)	12 (NA)	0.136	14 (5.5)	12 (5.5)	0.385
IVT	7 (21.2)	0	0.570	16 (45.7)	1 (33.3)	>0.99	14 (66.7)	20 (69.0)	>0.99
HT			0.972			0.498			0.250
HI-1, 2	5 (15.1)	0		7 (20.0)	1 (33.3)		2 (15.4)	7 (24.1)	
PH-1, 2	6 (18.2)	1 (20.0)		8 (22.8)	1 (33.3)		2 (9.5)	4 (13.7)	
mRS 0–2 at 3 months	16 (48.5)	2 (50.0)	>0.99	7 (20.0)	2 (66.7)	0.134	2 (9.5)	11 (37.9)	0.047*
Mortality	6 (18.2)	0	>0.99	9 (25.7)	0	>0.99	6 (28.6)	0	0.003

Abbreviations: IVT, Intravenous thrombolysis; HT, hemorrhagic transformation; HI, hemorrhagic infarction; PH, parenchymal hemorrhage; mRS, modified Rankin Scale.

*P<0.05 (OR, 6.059; 95% CI, 1.003–36.583) by multivariate logistic regression analysis, adjusted by age and initial NIHSS.

## Discussion

Our study showed data on stroke physicians' treatment preference for IAR in patients with acute ischemic stroke. It also highlighted interobserver variability in IAR decision for acute ischemic stroke, and the characteristics of low concordance about IAR decision. Small lesions on DWI and/or large mismatch on DWI-PWI had still good agreement of IAR decision. These results suggested that universally accepted criteria still need to define appropriate candidates for IAR in clinical practice. This study was not for clinical trials but real clinical practices.

It is certain that clinical decision making for the treatment of AIS should be based on scientific evidence, individual judgment of the clinician, and the opinion of the patients or their family. Although clinical decisions for AIS are generally made on the basis of these factors, our study has been designed because imaging studies have recently been implicated in clinical practice. Therefore, agreement on decisions for IAR between clinicians may not necessarily mean the correct decision for IAR in AIS.

We used consecutive patients admitted to a single stroke center in order to assess agreement of IAR decision among different stroke physicians. Unlike previous studies on agreement of diagnosis by imaging [22], [23], ours is unique to evaluate the agreement of IAR decision among stroke physicians in the different stroke centers. Previous investigators selected patients for thrombolysis according to their own imaging criteria [22], [24]. Therefore, it is noteworthy that our study analyzed imaging findings associated with IAR decision in different stroke physicians and investigated how differently the physicians selected IAR.

The ASPECTS scores on non-contrast CT, CT perfusion, and DWI were developed for risk stratification and prognostication in AIS patients [15]. Recently, Psychogios *et al*. have shown that CT perfusion parameters, as evaluated with ASPECTS, are more sensitive and specific than CT-ASPECTS in the prediction of favorable outcomes after endovascular treatment [25]. In particular, 2 CT perfusion scores, the CBV-ASPECTS and the discrepancy between CBV and CBF-ASPECTS (CBV-CBF ASPECTS), were significantly different between patients with favorable outcomes and those with poor clinical outcomes. These study results are consistent with ours. In addition, the correlation of ASPECTS mismatch and volumetric mismatch has been shown to be strong on CT and DWI [25], [26]. In the clinical studies, target mismatch was defined by using commercially unavailable automated software. Such automated software is limited because it has been used in only a few centers. However, the D-M ASPECTS, a semiquantitative measurement, could assess the infarct core and mismatch volume and can also be done without the need for volumetric software.

The overall agreement of IAR decision was poor among stroke physicians (kappa = 0.39). Only 51 of the 125 patients had complete concordance about IAR decision among physicians. All stroke physicians had good intra-rater agreement of IAR decision and this means that they had their own criteria for IAR decision. However, we demonstrated that D-M ASPECTS >7 had substantial agreement of IAR decision (kappa = 0.802). Previous studies defined target mismatch as a ratio between the volumes of hypoperfused tissues and an ischemic core of 1.8 or more [27]. Patients with target mismatch (a ratio between the volumes of critically hypoperfused tissue and the ischemic core of 1.8 or more, with an absolute difference of 15 mL or more; ischemic core volume of less than 70 mL; and less than 100 mL of tissue with a severe delay in bolus arrival) have an increased likelihood of favorable clinical outcomes after reperfusion [27]. However, target mismatch of another study was differently defined as a predicted infarct core of 90 ml or less and a proportion of predicted infarct tissue within the at-risk of 70% or less [12]. This discrepancy may significantly affect the results of clinical trials. Many investigators have recently attempted to include patients with much smaller infarct core volume in their clinical trials of IAR.

In addition, DWI-ASPECTS >6 showed also moderate agreement of IAR decision (kappa = 0.578). A previous study showed that DWI-ASPECTS >6 was an independent predictor of dramatic recovery at 7 days [28]. In contrast, patients with DWI-ASPECTS <6 is worse outcomes or symptomatic intracerebral hemorrhage [29], [30]. Briefly, a pretreatment DWI-ASPECTS of 5 or 6 might be a cutoff point of good outcomes in patients treated with IV thrombolysis, but not that of thrombolytic therapy. In MRI-based IAR decision, patients with initial infarct >70 cm^3^ had poor outcomes despite recanalization [31]. Our study suggested that a large DWI-MTT mismatch with a small DWI lesion could be highly agreeable findings for IAR performance among stroke physicians. The results of our study may support those of previous studies. Although we could not directly compare the target mismatch of previous studies to our D-M ASPECTS, a high score generally represented a large DWI-MTT mismatch. For example, a D-M ASPECTS of 9 means a DWI-ASPECTS of 9 or 10 and a MTT-ASPECTS of 0 or 1. In addition, group C with relatively good agreement of IAR performance had a high median D-M ASPECTS of 6.25.

However, there were no universally accepted criteria for defining the appropriate candidates for IAR. In our study, DWI-ASPECTS ≤5 had poor agreement of IAR decision. Considering previous results which patients with DWI-ASPECTS <6 had poor outcomes, they may have had good interobserver agreement of IAR decision. For example, since patients with a DWI-ASPECTS of 0 to 2 usually had large DWI lesions, it is thought that interobserver agreement was good about no IAR. However, interobserver agreement for a DWI-ASPECTS of 0 to 2 was poor (−0.042, −0.103, and 0.025, respectively). These results may be explained by the fact that physicians considered various variables including time, onset patterns, symptoms, and signal intensity of DWI, as well as lesion volume on DWI for the choice of IAR.

The equipoise of imaging criteria among physicians may be a gray zone of clinical practice for IAR. In addition, our study showed the characteristics of patients with clinical equipoise of IAR decision (group B). There were no significant differences in general characteristics between groups A and B (excluding patients with NIHSS <5 and occlusion of catheter inaccessible M2 and M3 segments). This may be caused by differences in the interpretation of clinical significance on DWI or PWI. Some physicians selected IAR on the basis of the presence and size of irreversible tissue, such as size of the DWI lesion. They also considered that ischemic lesions could be dangerous after IAR if it is sizable. Others selected IAR on the basis of the presence or size of reversible tissue, such as DWI-PWI mismatch. These results support the hypothesis that patients with small salvageable tissue may benefit more from recanalization than from non-recanalization. Many physicians in clinical practice will halt between the pros and cons of IAR in patients with moderate to large DWI lesions for IAR decision. Therefore, in moderate (or maybe large) DWI lesions with mismatch, agreement of IAR decision may not be good on the basis of the results of our study.

Clinical information, such as NIHSS scores and clinical presentations, could affect IAR decision. It was not an important factor for IAR decision whether it was clear onset or unclear onset in the study. However, group A showed a significantly higher frequency of patients with NIHSS <5 than groups B and C. Although acute mild stroke with proximal arterial occlusion can be considered to be at high risk of early neurological deterioration and poor outcomes [32]–[34], physicians are still conservative to such patients. Further studies with clinical parameters are needed to confirm our results.

The efficacy of IAR in acute ischemic stroke has yet to be determined [10]–[12]. The Mechanical Retrieval and Recanalization of Stroke Clots Using Embolectomy trials showed that a favorable penumbral pattern on neuroimaging did not identify patients who would differentially benefit from endovascular therapy [12]. In our study, however, patients who did not undergo IAR in group C had much lower frequency of favorable outcome at 3 months compared to those who did. This result suggests that it is unlikely to have favorable outcome if IAR is not performed on group C-like patients. Therefore, the results of our study suggest that MRI could play a key role in optimizing patient selection for IAR.

This study has some limitations. First, the size of DWI lesions and the area of hypoperfusion on MTT were not volumetrically evaluated in this study. Instead, we used the ASPECTS system. Although the ASPECTS have been shown to be feasible and relatively consistent with clinical outcomes and lesion volumes [26], [35], it has the disadvantage of evaluating DWI lesions and hypoperfused areas. Therefore, the results of our study should be interpreted with caution. Further studies are needed to confirm the clinical implications of DWI lesions and hypoperfused areas on MTT. Second, this study was conducted in limited areas in a single country. Although physicians working at various institutions participated in this study, there may be a selection bias because of restricted geographical limitations. Further studies with patients in wider areas are needed to confirm our results.

In summary, our study showed that higher DWI-ASPSECTS and D-M ASPECTS had better agreement of IAR decision among stroke physicians. However, there is still low agreement as to whether IAR should be performed in patients with moderate to large DWI lesions or not. Therefore, further study is warranted to have more acceptable imaging criteria for imaging based IAR decision in clinical practice.

## Supporting Information

Figure S1 **Flow diagram of patients' selection.**
(TIF)

Table S1 **The characteristics of participating centers (records of recent 2 years).**
(DOCX)
