# Supplementary figures and images for: To Do or Not to Do; Dilemma of Intra-Arterial Revascularization in Acute Ischemic Stroke
Source: PLoS One. 2014 Jun 6;9(6):e99261. doi: 10.1371/journal.pone.0099261 (PMC4048270; doi:10.1371/journal.pone.0099261)

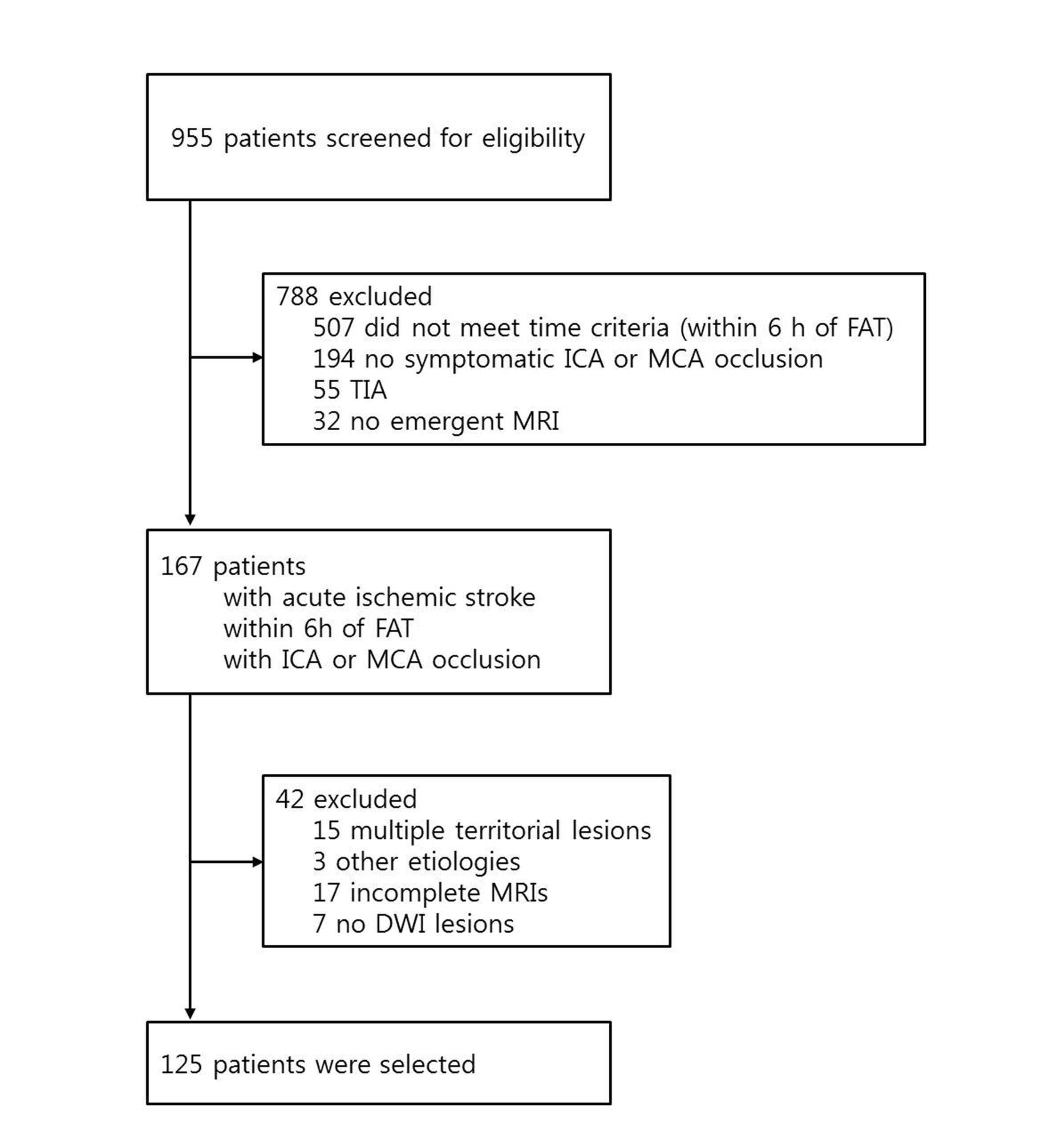

Supplement: Figure S1 — Flow diagram of patients' selection. (TIF) [file pone.0099261.s001.tif]
