# Supplementary material for: To Do or Not to Do; Dilemma of Intra-Arterial Revascularization in Acute Ischemic Stroke
Source: PLoS One. 2014 Jun 6;9(6):e99261. doi: 10.1371/journal.pone.0099261 (PMC4048270; doi:10.1371/journal.pone.0099261)
Supplement: Table S1 — The characteristics of participating centers (records of recent 2 years). (DOCX) [file pone.0099261.s002.docx]

Table S1. The characteristics of participating centers (records of recent 2 years)

|  | Center 1 (CNUH) | Center 2 (CUH) | Center 3 (JNUH) | Center 4 (WUH) |
| --- | --- | --- | --- | --- |
| Ischemic stroke patients (N) | 2229 | 1169 | 1395 | 1020 |
| IV-thrombolysis (N) | 343 | 140 | 125 | 156 |
| IAR (N) | 107 | 57 | 130 | 86 |
| Documented criteria for IAR | No | No | No | No |
| Decision tools | MRIs | CTA | MRIs or CTA | MRIs |
| Time for IAR availability | 7AM-11PM, everyday | All day, everyday | All day, everyday | All day, everyday |
| Stroke physicians (N)* | 5 | 2 | 2 | 2 |

*Stroke physicians: Dedicated specialists (Boards of Neurology) for ischemic stroke.
